# Novel Insights Into the Genetic Basis Between Cholelithiasis and Four Dietary Patterns

**DOI:** 10.1002/fsn3.71052

**Published:** 2025-10-12

**Authors:** Endong Zheng, Bangzhun Cai, Xiaowang Huang

**Affiliations:** ^1^ Department of Hepatobiliary Surgery The Affiliated Cangnan Hospital of Wenzhou Medical University Wenzhou China

**Keywords:** cholelithiasis, comorbidity gene, four dietary patterns, genetic overlap, genetic structure

## Abstract

Previous epidemiological evidence has demonstrated that cheese, dried fruit, oily fish, and raw vegetables intake is inversely associated with the risk of cholelithiasis. Nevertheless, the underlying genetic mechanisms responsible for this relationship remain elusive, warranting a genomic‐level investigation into the molecular pathways mediating these associations. To investigate potential genetic linkages, genome‐wide association study (GWAS) datasets pertaining to cholelithiasis and four specific dietary patterns (cheese, dried fruit intake, oily fish, and raw vegetables) were employed within a multi‐stage analytical framework. Initially, genome‐wide genetic correlations were assessed through a combination of linkage disequilibrium score regression, genetic covariance analysis, and high‐definition likelihood methodologies. Concurrently, local genetic variation analyses were conducted to pinpoint relevant genomic loci. Mendelian randomization (MR) was employed to assess causal effects. Subsequently, conditional/conjunctional false discovery rate (cond/conjFDR) approaches were utilized to evaluate the genetic overlap between cholelithiasis and dietary traits. Integration of conjFDR with multi‐trait analysis of GWAS (MTAG) facilitated the identification of shared genetic loci. Significant inverse genome‐wide genetic correlations were identified between cholelithiasis and all four dietary patterns. Analyses of local genetic variation revealed overlapping genetic signals across several chromosomal regions. The application of cond/conjFDR approaches provided further validation of genetic commonality between the traits. Integration of conjFDR with MTAG led to the successful identification and validation of several key shared genetic loci. This investigation represents the first genomic‐level analysis establishing genetic associations between cholelithiasis and cheese, dried fruit intake, oily fish, and raw vegetable intake. The elucidated shared genetic loci offer novel molecular insights supporting dietary strategies for cholelithiasis prevention.

## Graphical abstract

This study represents the first genomic‐level investigation into the genetic associations between cholelithiasis and four dietary patterns (cheese, dried fruit, oily fish, and raw vegetables). Genome‐wide analysis revealed significant overall and local negative genetic correlations between cholelithiasis and these four dietary patterns. Shared genetic loci were successfully identified using conditional/conjunctional false discovery rate (cond/conjFDR) and multi‐trait analysis of GWAS (MTAG) methods. These findings offer new molecular insights for preventing cholelithiasis and support dietary intervention strategies.

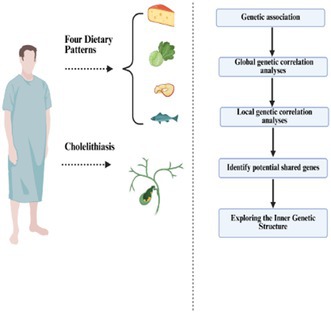

## 1 Introduction

Cholelithiasis is defined as a pathological condition characterized by the formation of stones within the gallbladder or bile ducts resulting from metabolic disturbances in bile constituents (Wang et al. 2024). It represents one of the most prevalent disorders of the digestive system, affecting approximately 10%–20% of the global adult population (Shabanzadeh 2018). Clinical manifestations are varied, with hallmark symptoms including colicky pain localized to the right upper quadrant, typically precipitated by the intake of high‐fat meals, and frequently accompanied by nausea, vomiting, and pain radiating to the right shoulder (Reshetnyak 2012). Evidence suggests that asymptomatic gallstone carriers progress to symptomatic states at an estimated annual rate of 1%–4% (Littlefield and Lenahan 2019). Upon symptom onset, approximately 35%–50% of affected individuals experience recurrent pain or complications, such as cholecystitis (10%–20%), cholangitis (5%–15%), and pancreatitis (5%) (Lammert et al. 2016). Etiological investigations have indicated that, beyond immutable intrinsic determinants (e.g., sex‐based differences, aging stages, and genetic predisposition), various extrinsic factors substantially contribute to the development of cholelithiasis. These include dietary composition, lifestyle practices, and environmental exposures (Pak and Lindseth 2016; Di Ciaula et al. 2018). Epidemiological evidence has proposed that certain traditional dietary elements—particularly cheese, dried fruit intake, oily fish, and raw vegetables—may confer protective benefits against gallstone formation, likely attributable to their distinct nutritional profiles and bioactive compounds (Xie et al. 2024; Gutiérrez‐Díaz et al. 2018; Wang et al. 2022). Specifically, it has been suggested that unsaturated fatty acids from oily fish, antioxidant compounds and dietary fiber from raw vegetables and dried fruits, as well as bioactive peptides and calcium from cheese, may collectively improve cholesterol metabolism, modulate gut microbiota, and reduce oxidative stress, thereby lowering the risk of gallstone development (Li et al. 2022). Given the potential confounding effects arising from interrelated dietary variables in conventional observational studies, elucidating the inherent molecular linkages between cholelithiasis susceptibility and the intake of these four dietary patterns through genomic methodologies may yield more accurate and mechanistic insights.

Advancements in genome‐wide association study (GWAS) technology have facilitated the identification of genetic variation spectra associated with both cholelithiasis and specific dietary habits (e.g., cheese, dried fruit intake, oily fish, and raw vegetable intake), thereby offering scientific foundations for interpreting their potential genetic interrelations. In recent years, the development of innovative statistical genetics algorithms has enabled cross‐phenotype genetic analyses between complex traits. These methodologies have proven effective in uncovering genetic interaction patterns among diverse conditions, including the confirmation of genetic overlap between schizophrenia and constipation (Luo et al. 2025), the identification of shared genetic loci linking alcohol and cheese intake with inflammatory bowel disease (Huang and Yuan 2025), and the clarification of genetic structural associations between testosterone levels and polycystic ovary syndrome (Sun et al. 2024). The present study was designed to systematically investigate the genetic interaction characteristics between cholelithiasis susceptibility and the aforementioned four dietary patterns by employing these validated statistical genetic approaches.

A multivariate genetic statistical analysis framework was established in the present study to characterize genetic interaction patterns among the target phenotypes. At the genome‐wide level, linkage disequilibrium score regression (LDSC) (Bulik‐Sullivan et al. 2015), genetic covariance analyzer (GNOVA) (Lu et al. 2017), and the high‐dimensional likelihood model (HDL) (Ning et al. 2020) were implemented to quantify global genetic correlations, while local genetic variation analysis (LAVA) (Werme et al. 2022) was incorporated to identify region‐specific genetic interaction signals. Mendelian randomization (MR) analysis was conducted to infer potential causality (Emdin et al. 2017). To achieve precise localization of shared genetic variants, a combined strategy involving conditional/conjunctional false discovery rate (cond/conjFDR) methods (Smeland et al. 2020) and multi‐trait analysis of GWAS (MTAG) (Turley et al. 2018) was utilized. Within this framework, the conjFDR approach not only assesses the extent of polygenic interactions but also facilitates the efficient detection of shared genetic risk loci (Smeland et al. 2020). MTAG, as a widely adopted computational tool, demonstrates notable efficacy in identifying gene loci associated with comorbid traits (Turley et al. 2018). This comprehensive analytical strategy contributes to the elucidation of the potential genetic correlation network linking cholelithiasis and the four dietary patterns.

## 2 Methods and Materials

### 2.1 GWAS Data

The dietary behavior genomic data employed in this study were retrieved from the IEU GWAS data repository (https://gwas.mrcieu.ac.uk/). Specifically, genome‐wide association data for four dietary patterns were included: cheese intake (ID: ukb‐b‐1489), dried fruit intake (ID: ukb‐b‐16576), oily fish intake (ID: ukb‐b‐2209), and raw vegetable intake (ID: ukb‐b‐1996). The cholelithiasis phenotype dataset was obtained from the 12th release of the FinnGen project, designated as “K11_CHOLELITH” (Kurki et al. 2023). Population characteristics corresponding to each dataset are presented in Table 1.

**TABLE 1:** Date sources.

Phenotypes	Phenotypic code	Ancestry
Cholelithiasis	K11_CHOLELITH	European
Cheese intake	ukb‐b‐1489	European
Dried fruit intake	ukb‐b‐16,576	European
Oily fish intake	ukb‐b‐2209	European
Raw vegetable intake	ukb‐b‐1996	European

**FIGURE 1:**
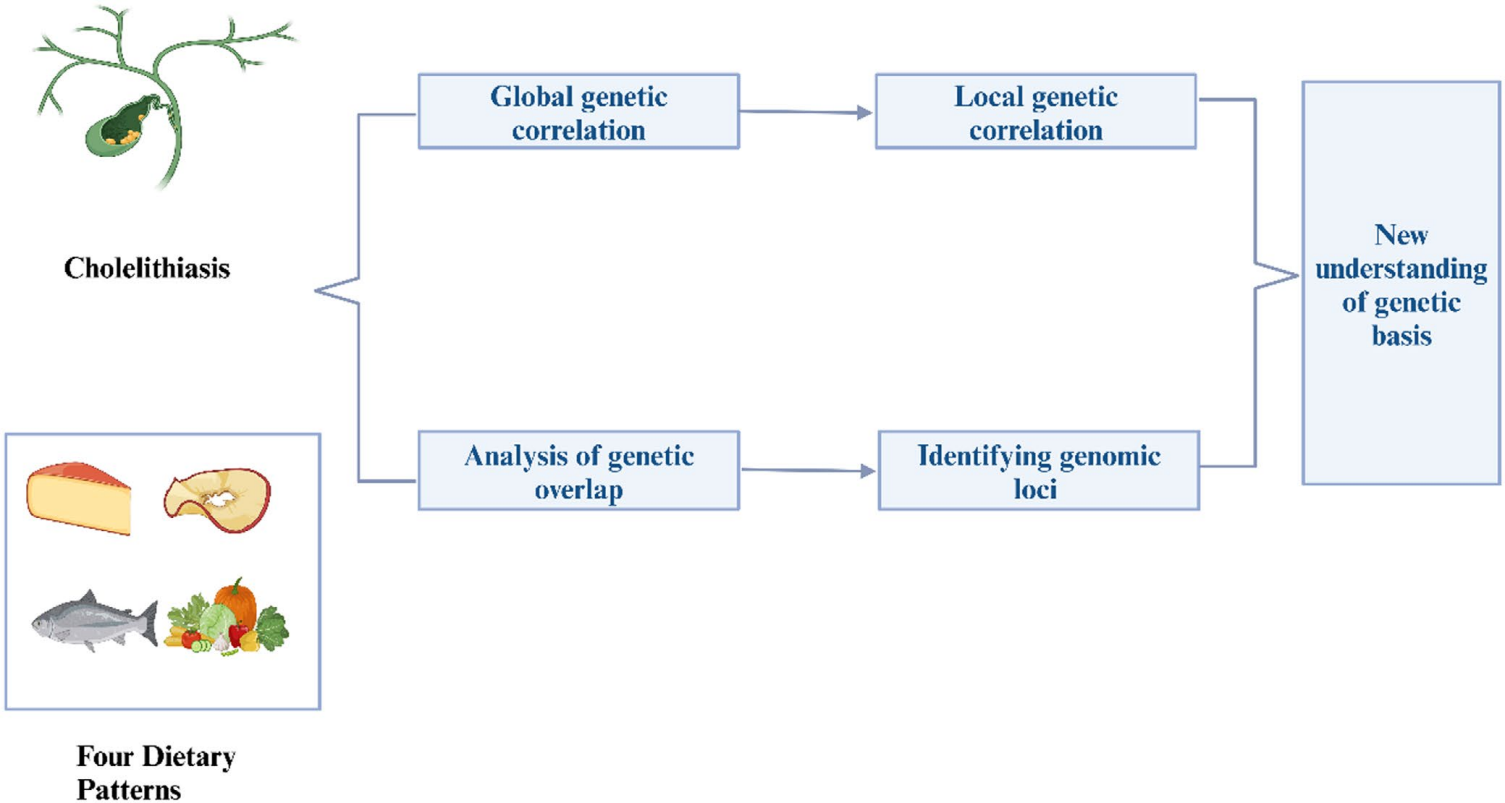
Flowchart of the study. The diagram was created with BioRender.

To ensure the reliability of the analytical outcomes, rigorous data screening criteria were formulated and applied. The genetic variants included were required to satisfy the following conditions: (Wang et al. 2024) presence in the reference sequence from Phase 3 of the 1000 Genomes Project; (Shabanzadeh 2018) minor allele frequency (MAF) exceeding 0.01 in European populations; and (Reshetnyak 2012) restriction to biallelic variants only. All variants were annotated based on their chromosomal positions using the human reference genome hg19/GRCh37, and those lacking rsID information or containing duplicate rsIDs were excluded. Considering population‐specific genetic backgrounds, only data derived from individuals of European ancestry were analyzed in this study. The complete research workflow is illustrated in Figure 1.

### 2.2 Global Genetic Correlation Analyses

The LDSC analytical workflow for evaluating genome‐wide genetic correlation comprises several sequential steps, including data preprocessing, linkage disequilibrium (LD) score computation, regression modeling, genetic correlation estimation, error correction, and interpretation of results (Bulik‐Sullivan et al. 2015). Initially, LD scores are calculated for high‐quality single nucleotide polymorphisms (SNPs). Thereafter, weighted least squares (WLS) regression is applied to examine the association between SNP effect sizes (*Z*‐scores) and LD scores, enabling the inference of genetic covariance (), while standard errors are estimated using the Jackknife resampling technique. Genetic correlation () is then derived by normalizing the estimated genetic covariance. To mitigate bias resulting from sample overlap, a constrained intercept method is employed, accompanied by population structure adjustments (e.g., principal component analysis) to reduce false‐positive signals. The resulting genetic correlations are visualized in the form of a matrix, allowing the characterization of genetic relationships across traits. LDSC operates independently of individual‐level genotype data and provides robust control for sample overlap effects, making it a widely adopted approach in genetic epidemiological research.

GNOVA is a method designed to estimate genetic covariance based on GWAS summary statistics and is recognized for its superior precision and statistical power relative to LDSC while also supporting analysis stratified by functional annotation (Lu et al. 2017). The process begins with the screening of high‐quality SNPs and the calculation of LD information, followed by the establishment of a linear model that permits SNP effects to vary across distinct functional genomic regions. GNOVA applies moment‐based estimation to derive linear equations and resolves genetic covariance parameters using various weight matrices. Block jackknife methods are subsequently used to estimate standard errors and adjust for sample overlap. In addition, GNOVA enables stratified genetic covariance analysis based on functional genomic annotations or MAF, facilitating the identification of shared genetic architecture underlying complex traits. Ultimately, GNOVA produces genetic correlation matrices, delivering more accurate genetic covariance estimates with enhanced biological interpretability, thereby serving as a robust analytical tool in the genetic investigation of complex diseases.

HDL is a full‐likelihood‐based method for estimating genetic correlation that efficiently extracts LD information from GWAS summary statistics and exhibits advantages over LDSC in terms of enhanced estimation precision and statistical efficiency (Ning et al. 2020). The procedure begins by calculating GWAS *Z* statistics for SNPs and establishing a genetic covariance model based on genome‐wide LD matrices. Genetic correlation is estimated using maximum likelihood, while eigenvalue decomposition is applied to regularize the LD matrix, thereby alleviating computational complexity. In addition, block jackknife methods are employed to compute standard errors, ensuring the robustness of the estimates. Relative to LDSC, this method reduces the variance in genetic correlation estimates by approximately 60%, corresponding to a 2.5‐fold increase in effective sample size, and is thus particularly well suited for traits characterized by low heritability or binary outcomes.

### 2.3 Local Genetic Correlation Analyses

LAVA is a computational tool used for local genetic correlation (local rg) analysis that identifies shared genetic signals between phenotypes and characterizes conditional genetic relationships (Werme et al. 2022). The process begins with the calculation of LD matrices based on GWAS summary statistics, followed by the estimation of SNP joint effects through multivariate linear regression to ensure consistency in effect directions across phenotypes. Upon screening for local genetic signals, LAVA utilizes moment‐based estimation to compute genetic variance matrices, from which local genetic correlations are derived. Simulation testing is subsequently conducted to evaluate statistical significance. In addition, LAVA supports partial correlation analysis and multivariate regression modeling, facilitating the investigation of complex genetic regulatory mechanisms and pleiotropic hotspots. To ensure estimate robustness, block jackknife methods are applied for error correction, enabling the resolution of heterogeneous genetic patterns across distinct genomic regions. In contrast to global genetic correlation methods, LAVA offers more precise detection of concealed local genetic associations, thereby providing deeper insights into the genetic architecture of complex diseases.

### 2.4 Mendelian‐Randomization Analysis

This study applied a bidirectional two‐sample MR approach to examine the causal associations between cholelithiasis and four dietary patterns. The entire analysis strictly adhered to the three core assumptions of MR (Emdin et al. 2017). In the genome‐wide analysis, we performed stringent variant selection and linkage disequilibrium (LD) clumping, with the thresholds set as follows: genome‐wide significance *p* < 5 × 10^−8^, LD parameter *r* ^2^ = 0.001, and genomic distance kbp = 10,000.

To ensure the robustness and reliability of the results, we conducted a series of sensitivity analyses, including tests for horizontal pleiotropy (Burgess and Thompson 2017; Verbanck et al. 2018), heterogeneity analyses (Luo et al. 2023), and leave‐one‐out validation (Burgess et al. 2017) as quality control measures. All MR analyses were performed in the R environment, primarily using the TwoSampleMR package (available at: https://mrcieu.github.io/TwoSampleMR/) and the MR‐PRESSO package (available at: https://github.com/rondolab/MR‐PRESSO) for data analysis and causal inference.

### 2.5 Cond/conjFDR Analysis

cond/conjFDR are statistical approaches employed in GWAS data analysis to enhance the detection of genetic signals and identify shared genetic loci across distinct traits (Smeland et al. 2020). The condFDR method operates within an empirical Bayesian framework, adjusting the GWAS statistical outcomes of a primary trait by leveraging SNP association information from auxiliary traits. This adjustment refines the ranking of statistical tests and reduces the false discovery rate (FDR). The process begins with the preparation of two or more independent GWAS datasets to ensure alignment of SNP effect directions, followed by standard quality control procedures and LD information computation. During condFDR estimation, conditional quantile‐quantile (Q‐Q) plots are generated to depict SNP enrichment patterns. Subsequently, FDR is computed through stratification based on auxiliary trait *p*‐values, and a binary FDR lookup table is constructed to increase GWAS discovery efficiency.

conjFDR extends the condFDR framework to detect shared SNPs that exert simultaneous effects on two traits. The calculation involves independently computing condFDR for each trait and selecting the larger value as the final conjFDR score, thereby yielding a conservative estimate of shared genetic signals. This approach facilitates the identification of common genetic variants even in cases where the overall genetic correlation is low. In addition, genomic inflation factors are applied to correct for biases arising from population structure or sample overlap, while decorrelation procedures (*r* ^2^ < 0.1) are used to mitigate the confounding effects of LD structure. Ultimately, condFDR enhances the discovery power of GWAS, whereas conjFDR enables the detection of shared genetic architectures between traits, contributing to the investigation of comorbidity mechanisms and functional genomic annotations, and offering considerable applicability in genetic research on complex traits and diseases.

### 2.6 Cross‐Trait Meta‐Analysis

MTAG enhances statistical power and produces trait‐specific SNP effect estimates by integrating GWAS summary statistics across multiple related traits (Turley et al. 2018). The procedure begins with the application of LDSC to estimate the GWAS error covariance matrix Σ, thereby accounting for potential biases introduced by sample overlap and population structure. Following this, the variance–covariance matrix Ω of SNP effects is derived using the method of moments, capturing the genetic correlations among traits. Based on these components, MTAG employs WLS to compute SNP effect estimates and adjusts standard errors and *P*‐values to ensure comparability with single‐trait GWAS results. The final output includes refined SNP effect estimates suitable for downstream applications such as biological annotation, gene enrichment analysis, and polygenic risk score development. Compared to single‐trait GWAS, MTAG substantially increases the number of identified loci and enhances the precision of effect size estimates. This method is particularly effective in improving detection efficiency for genetic variants associated with highly correlated traits.

For the genetic variant loci identified through the cond/conjFDR method and MTAG analysis, systematic gene annotation was conducted using the SNP2GENE functional module within the Functional Mapping and Annotation (FUMA) platform (Watanabe et al. 2017).

## 3 Results

### 3.1 Global Genetic Correlation

Through comprehensive systemic genetic analysis, significant genetic correlations were identified between cholelithiasis and four distinct dietary patterns. LDSC analysis revealed a significant inverse genetic correlation between cholelithiasis and cheese intake (Rg = −0.20, *p* = 6.93e‐08). Comparable negative associations were also detected for the other three dietary factors: dried fruit intake (Rg = −0.16, *p* = 2.73e‐10), oily fish (Rg = −0.14, *p* = 2.74e‐05), and raw vegetable intake (Rg = −0.12, *p* = 8.72e‐06) (Table 2).

**TABLE 2:** The genetic correlation and sample overlap correlation between cholelithiasis and four dietary patterns.

Trait1	Trait2	LSDC‐Rg	LSDC‐P	GNOVA‐Rg	GNOVA‐P	HDL‐Rg	HDL‐P	*Z*‐score‐*p*
Cheese intake	Cholelithiasis	−0.20	6.93e‐08	−0.13	2.82e‐05	−0.22	2.02e‐08	0.018
Dried fruit intake	Cholelithiasis	−0.16	2.73e‐10	−0.14	2.44e‐13	−0.18	3.13e‐08	0.012
Oily fish intake	Cholelithiasis	−0.14	2.74e‐05	−0.14	5.94e‐06	−0.15	6.48e‐09	0.020
Raw vegetable intake	Cholelithiasis	−0.12	8.72e‐06	−0.12	8.83e‐11	−0.11	1.46e‐05	0.026

*Note:* Rg: Correlation between two traits, rg ranges from −1 to 1, and the closer the value is to 1 or −1, the stronger the correlation is (plus or minus represents positive and negative correlation); *p*: The *p*‐value of genetic correlation; *Z*‐score‐*p*: The *p*‐value of calculate potential sample overlap scores.

Abbreviations: HDL, high‐dimensional likelihood; LDSC, linkage disequilibrium score regression.

To assess the reliability of the results, two independent methods—GNOVA and HDL—were utilized for cross‐validation. The outcomes of these supplementary analyses exhibited strong concordance with the primary findings, thereby reinforcing the stability and reproducibility of the identified genetic associations between cholelithiasis and the four aforementioned dietary patterns (Table 2). This observation offers a novel perspective for investigating the potential shared genetic mechanisms that underlie the relationship between cholelithiasis and dietary behaviors.

### 3.2 Local Genetic Correlation

LAVA identified intricate patterns of association between cholelithiasis and the four dietary patterns at the chromosomal level. The findings demonstrated that multiple chromosomal regions exhibited significant correlations with both cholelithiasis and various dietary factors.

**FIGURE 2:**
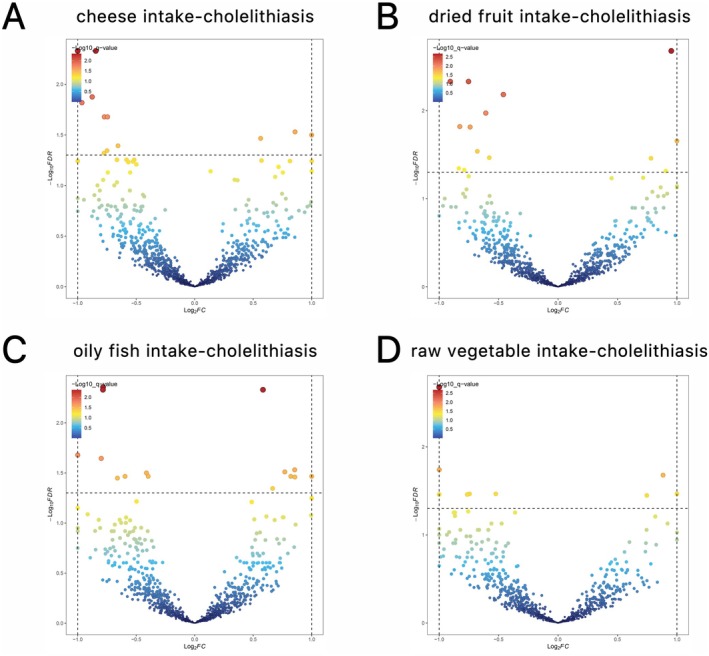
LAVA analysis of four dietary patterns and cholelithiasis. The dashed line indicates the expected line with a correction *p* of 0.05. (A) Local genetic correlation between cheese intake–cholelithiasis. (B) Local genetic correlation between dried fruit intake–cholelithiasis. (C) Local genetic correlation between oily fish intake–cholelithiasis. (D) Local genetic correlation between raw vegetable intake–cholelithiasis. LAVA, local variant association.

Twelve chromosomal regions exhibiting significant correlations between cholelithiasis and cheese intake were identified, comprising three positively correlated regions (located on chromosomes 5, 8, and 13) and nine negatively correlated regions (located on chromosomes 2, 6, 8, 11, 12, 16, and 18) (Figure 2A, Table S1).

Regarding dried fruit intake, fourteen chromosomal loci exhibiting significant correlations were identified, comprising four positively correlated regions (located on chromosomes 2, 10, 14, and 15) and ten negatively correlated regions (located on chromosomes 1, 4, 5, 7, 10, 11, 12, 17, and 19) (Figure 2B, Table S2).

For oily fish intake, fifteen markedly correlated chromosomal regions were detected, including seven with positive correlations (distributed across chromosomes 2, 5, 6, 7, 8, 13, and 16) and eight with negative correlations (distributed across chromosomes 2, 6, 7, 10, 15, and 19) (Figure 2C, Table S3).

Raw vegetable intake was associated with nine chromosomal regions demonstrating significant correlations, consisting of three positively correlated regions (located on chromosomes 1, 10, and 16) and six negatively correlated regions (located on chromosomes 2, 6, 13, and 21) (Figure 2D, Table S4).

### 3.3 Mendelian Randomization

A bidirectional MR analysis was conducted to examine the causal relationship between cholelithiasis and four dietary habits. When dietary habits were considered as exposures and cholelithiasis as the outcome, higher intake of cheese and dried fruits was inversely associated with the risk of cholelithiasis, consistent with findings from previous MR studies (Xie et al. 2024; Yang et al. 2024). In contrast, no significant causal associations were observed for oily fish or raw vegetable intake. In the reverse analysis, where cholelithiasis was treated as the exposure and dietary habits as the outcomes, no significant causal effects were identified.

Across all MR analyses, no evidence of horizontal pleiotropy was detected, indicating the validity and reliability of the selected instrumental variables. Furthermore, all F‐statistics exceeded the conventional threshold of 10, suggesting sufficient instrument strength and minimizing the potential for weak instrument bias, thereby reinforcing the robustness of the causal estimates (Table S5). Leave‐one‐out sensitivity analyses demonstrated a relatively consistent distribution of SNP effects, with no outlier variants detected.

### 3.4 conjFDR Analysis Identifies Shared Genomic Loci Between Two Traits

**FIGURE 3:**
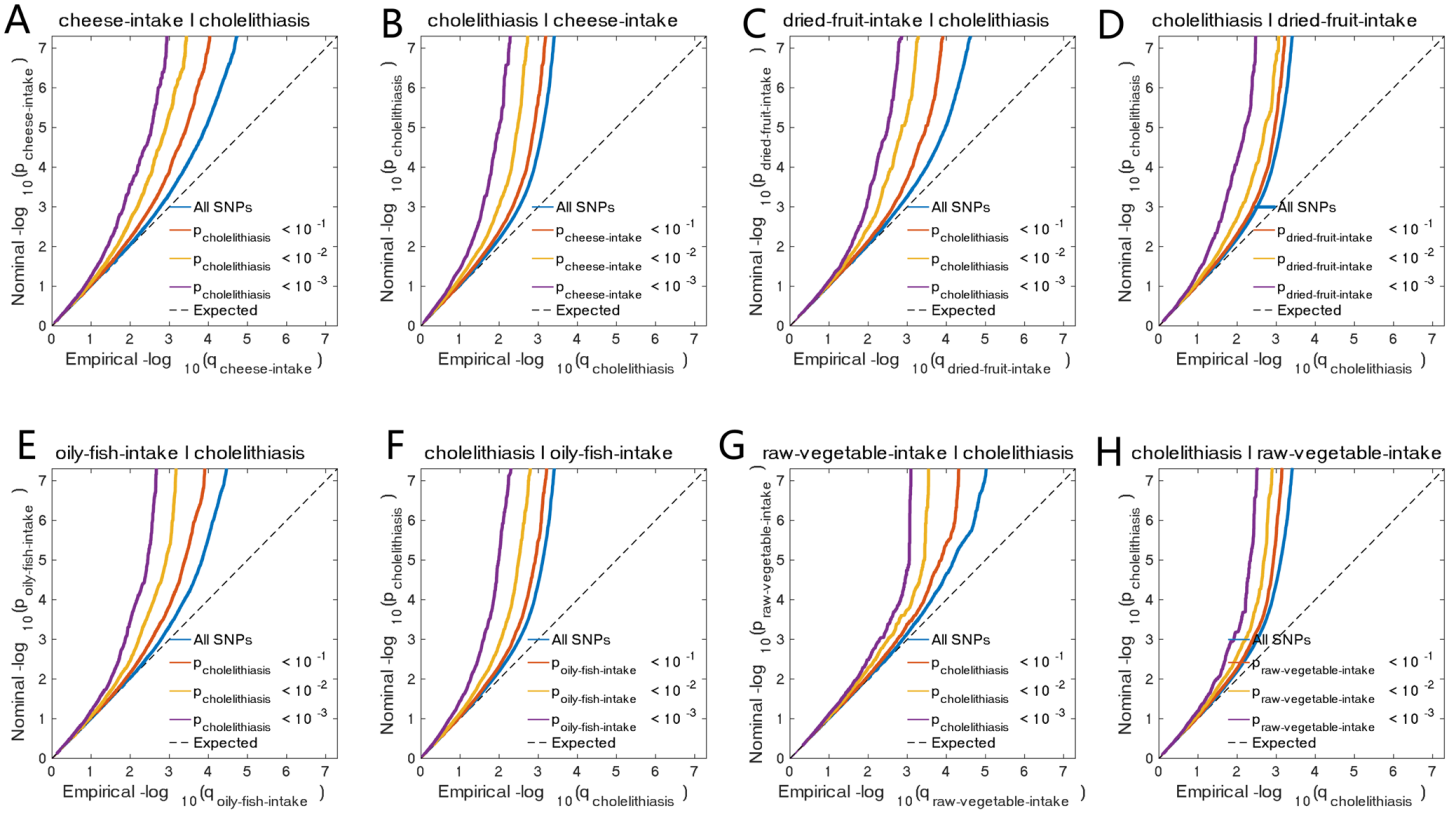
Conditional quantile‐quantile plot. The dashed line indicates the expected line under the null hypothesis, and the deflection to the left indicates the degree of pleiotropic enrichment. (A) Cheese intake–cholelithiasis. (B) Cholelithiasis–cheese intake. (C) Dried fruit intake–cholelithiasis. (D) Cholelithiasis–dried fruit intake. (E) Oily fish intake–cholelithiasis. (F) Cholelithiasis–oily fish intake. (G) Raw vegetable intake–cholelithiasis. (H) Cholelithiasis–raw vegetable intake.

The results of Q‐Q plot analysis revealed significant genetic correlations between cholelithiasis and the four dietary patterns at the genomic level (Figure 3). The analytical findings indicated that as disease‐associated signals became more pronounced, the correlation values of the corresponding dietary behaviors demonstrated systematic leftward deviations. This deviation pattern not only corroborated the presence of genetic associations among these traits but also implied the potential existence of shared disease susceptibility loci, thereby offering empirical support for the investigation of underlying shared molecular mechanisms.

**FIGURE 4:**
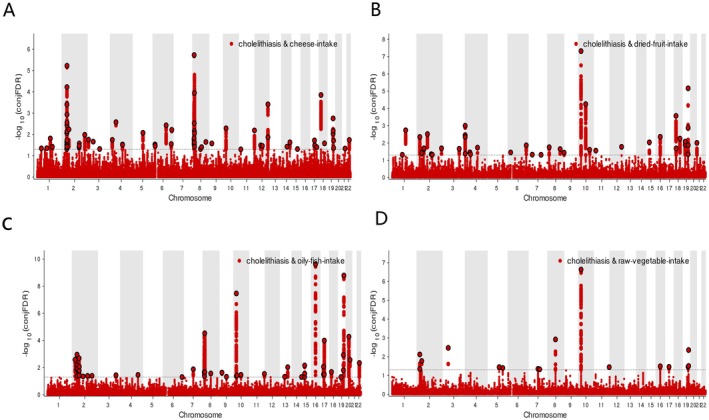
ConjFDR Manhattan plot of four dietary patterns and cholelithiasis. (A) Cholelithiasis‐cheese intake. (B) Cholelithiasis‐dried fruit intake. (C) Cholelithiasis‐oily fish intake. (D) Cholelithiasis‐raw vegetable intake. The statistically significant causality is defined to be conjFDR < 0.05.

The *Z*‐score statistical analysis indicated that no statistically significant sample overlap was observed between cholelithiasis and the four dietary habits (all comparisons, *p* < 0.05) (Table 2). By applying the conjFDR method for comprehensive genomic association analysis, a total of 80 pleiotropic genetic variants, corresponding to 68 genes, were identified between cholelithiasis and cheese intake at a conjFDR threshold of < 0.05 (Figure 4A, Table S6).

Further analysis revealed that 42 genetic variants, mapped to 40 genes, were shared between cholelithiasis and dried fruit intake (Figure 4B, Table S7); 51 loci, corresponding to 48 genes, were shared with oily fish intake (Figure 4C, Table S8); and 16 shared SNPs, involving 15 genes, were identified in association with raw vegetable intake (Figure 4D, Table S9). These genome‐wide findings provide a basis for elucidating the shared genetic architecture underlying cholelithiasis pathogenesis and specific dietary behaviors.

### 3.5 MTAG

**FIGURE 5:**
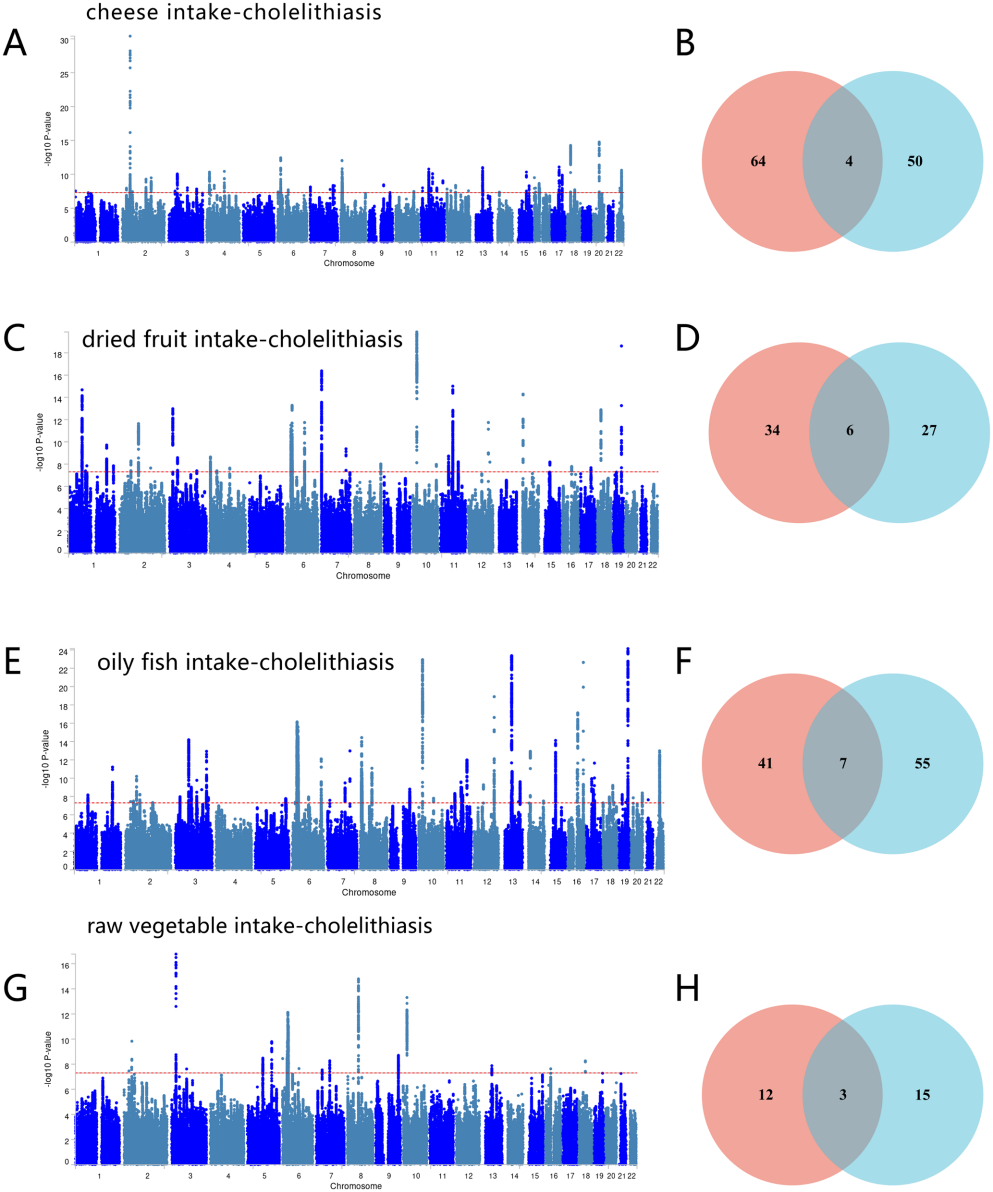
(A) Manhattan map of genetic risk loci for cheese intake and cholelithiasis by MTAG. (B) Intersection gene map of cheese intake and cholelithiasis after conjfdr and MTAG analysis. (C) Manhattan map of genetic risk loci for dried fruit intake and cholelithiasis intake by MTAG. (D) Intersection gene map of dried fruit intake and cholelithiasis after conjfdr and MTAG analysis. (E) Manhattan map of genetic risk loci for oily fish intake and cholelithiasis by MTAG. (F) Intersection gene map of oily fish intake and cholelithiasis after conjfdr and MTAG. (G) Manhattan map of genetic risk loci for raw vegetable intake and cholelithiasis by MTAG. (H) Intersection gene map of raw vegetable intake and cholelithiasis after conjfdr and MTAG.

The FUMA system was employed to conduct a comprehensive analysis of the MTAG output results, thereby identifying 54 shared genetic variant loci associated with both cholelithiasis and cheese intake (Figure 5A, Table S10). Notably, utilizing a bidirectional validation strategy that integrated conjFDR with MTAG analysis, four core genes were effectively identified: RP11‐89K21.1, CSRNP3, RP11‐362I1.1, and RP11‐115 J16.1 (Figure 5B, Table 3).

**TABLE 3:** Overlapping genes identified by conjFDR and MTAG analyses between gallstone disease and four dietary habits.

Trait1	Trait2	Comorbid genes	Function
Cheese intake	Cholelithiasis	RP11‐89 K21.1	Intergenic
Cheese intake	Cholelithiasis	CSRNP3	Intronic
Cheese intake	Cholelithiasis	RP11‐362I1.1	Intergenic
Cheese intake	Cholelithiasis	RP11‐115 J16.1	ncRNA_intronic
Dried fruit intake	Cholelithiasis	BCL11A	Intronic
Dried fruit intake	Cholelithiasis	RP11‐103 J17.1	Intergenic
Dried fruit intake	Cholelithiasis	TET2	Upstream
Dried fruit intake	Cholelithiasis	WSCD2	Intronic
Dried fruit intake	Cholelithiasis	SEMA6D	Intronic
Dried fruit intake	Cholelithiasis	APOE	Exonic
Oily fish intake	Cholelithiasis	GCKR	Exonic
Oily fish intake	Cholelithiasis	AC098824.6	Intergenic
Oily fish intake	Cholelithiasis	AC007131.1	ncRNA_intronic
Oily fish intake	Cholelithiasis	TEX41	ncRNA_intronic
Oily fish intake	Cholelithiasis	SEMA6D	Intronic
Oily fish intake	Cholelithiasis	FTO	Intronic
Oily fish intake	Cholelithiasis	IKZF3	Intronic
Raw vegetable intake	Cholelithiasis	KDM3B	Intronic
Raw vegetable intake	Cholelithiasis	GCKR	Exonic
Raw vegetable intake	Cholelithiasis	SKIDA1	UTR5

Abbreviations: ConjFDR, conjunctional false discovery rate; MTAG, multi‐trait analysis of GWAS.

In the MTAG analysis of dried fruit intake, 33 shared genetic susceptibility loci were identified as being associated with both dried fruit intake and cholelithiasis (Figure 5C, Table S11). Subsequent cross‐validation employing the cond/conjFDR method validated six genes—BCL11A, RP11‐103J17.1, TET2, WSCD2, SEMA6D, and APOE—to exhibit statistically significant associations across both independent analytical approaches (Figure 5D, Table 3).

The MTAG analysis of oily fish intake resulted in the identification of 62 shared genetic variants associated with cholelithiasis (Figure 5E, Table S12). Of these, seven genes—GCKR, AC098824.6, AC007131.1, TEX41, SEMA6D, FTO, and IKZF3—were independently validated by means of the conjFDR method (Figure 5F, Table 3).

In relation to raw vegetable intake, 18 associated loci were identified using the MTAG method (Figure 5G, Table S13). Upon cross‐validation, the genes KDM3B, GCKR, and SKIDA1 were validated to possess statistically significant shared genetic foundations with both cholelithiasis and raw vegetable intake behavior (Figure 5H, Table 3).

## 4 Discussion

In this study, GWAS technology was employed to examine the genetic associations between cholelithiasis and four dietary patterns. At the genomic level, statistically significant negative genetic correlations were detected among these factors. Regional genetic association analysis revealed multiple chromosomal segments exhibiting notable association signals. The existence of genetic overlap among these phenotypes was further supported through Q‐Q plot analysis. By integrating conjFDR and MTAG as dual validation approaches, several putative shared genetic loci were successfully identified. Collectively, these findings offer a novel genetic perspective on the mechanisms through which cheese, dried fruit intake, oily fish, and raw vegetables may contribute to the prevention and management of cholelithiasis, thereby enriching the understanding of diet‐related genetic factors in disease risk.

Recent studies have confirmed that higher cheese intake is markedly associated with a reduced risk of cholelithiasis. The calcium content in cheese has been shown to bind with bile acids and fatty acids, resulting in the formation of insoluble calcium soaps and thereby lowering the concentration of free cholesterol in the gallbladder (Wang et al. 2009). Findings from a survey conducted by Alyahyawi et al. (2023) indicate that habitual cheese intake may exert a protective effect. Casein glycomacropeptide in cheese has been identified by UHPLC–MS/MS, providing molecular evidence for its potential role in cholesterol regulation (Chen et al. 2024). Moreover, recent Mendelian randomization analyses have further substantiated this association by suggesting a causal link between increased frequency of cheese intake and a diminished risk of gallstone formation (Yang et al. 2024). Cheese intake may also attenuate the influence of ABCG5/ABCG8 gene polymorphisms on the development of cholelithiasis, given that these genes are involved in the regulation of cholesterol transport from the liver to bile (Krawczyk et al. 2011). In addition, evidence has indicated that cheese intake exhibits a more pronounced protective effect in carriers of PNPLA3 risk variants, potentially mitigating genetic susceptibility to cholelithiasis (Lammert et al. 2016). Research concerning the four genes validated in the present study remains limited in the context of their relationship with cheese intake and cholelithiasis. These genes may participate in the preventive mechanism and merit further exploration in subsequent investigations.

Research has established that dried fruit intake, which is abundant in dietary fiber, serves a pivotal function in the prevention and treatment of cholelithiasis (Alasalvar et al. 2023). Dietary fiber has been shown to effectively lower cholesterol levels by facilitating fecal neutral sterol excretion and inhibiting the transformation of primary bile acids into secondary bile acids (Hu et al. 2022; Arjmandi et al. 1992). In vitro experiments have demonstrated that various types and forms of raisins are capable of forming complexes with bile acids (Camire and Dougherty 2003). Recent Mendelian randomization analysis has further validated the causal association between dried fruit and reduced cholelithiasis risk at the genetic level (Xie et al. 2024). Dried fruits are rich in polyphenols, which suppress energy intake, regulate gastrointestinal hormones, promote beneficial bacteria, and reduce lipid peroxidation and liver enzyme markers, thereby lowering the risk of gallstone disease (Dong et al. 2022). These findings are consistent with the conclusions drawn in the current study. Among the identified genes, APOE and WSCD2 warrant particular attention. APOE serves a pivotal function in the pathogenesis of cholelithiasis. As one of the principal components of lipoproteins, APOE is involved in cholesterol and lipid transport, thereby influencing lipid metabolism and bile composition (Abu Abeid et al. 2002). Evidence has indicated that APOE gene polymorphisms are strongly associated with cholelithiasis risk, with ε4 allele carriers demonstrating a markedly elevated risk of cholesterol gallstone formation (Hasegawa et al. 2003; Dixit et al. 2006). Júnior S et al. confirmed that APOE directly affects the physicochemical properties of bile by regulating cholesterol transport from hepatocytes into bile (Pinheiro‐Júnior et al. 2012). Notably, one study examining dietary habits and cholelithiasis also recognized the important role of APOE in this relationship (Jin et al. 2024). Intake of dried fruit rich in unsaturated fatty acids has been reported to markedly upregulate APOE gene expression (average increase of 23.7%, *p* = 0.008), thereby enhancing reverse cholesterol transport capacity (Lankinen et al. 2014). The WSCD2 gene, newly identified as a susceptibility gene for cholelithiasis, appears to serve a distinct function in gallstone formation. Joshi et al. initially reported a significant association between WSCD2 and cholelithiasis through GWAS (*p* = 2.15 × 10^−9^) (Joshi et al. 2016). From a functional standpoint, the protein encoded by WSCD2 contains WSC domains, which are commonly involved in cell wall integration and extracellular signal transduction. In vitro studies conducted by Rodriguez et al. (2016) demonstrated that WSCD2 may influence β‐glucuronidase activity in gallbladder epithelial cells, thereby affecting bile acid metabolism. Furthermore, findings by Jiang et al. (2008), using conditional gene knockout mouse models, revealed that liver‐specific WSCD2 deficiency markedly alters mucin secretion in bile and modifies its physicochemical characteristics, suggesting a potential role in gallstone formation through the regulation of bile composition.

The association between oily fish intake and the prevention of cholelithiasis has been extensively supported by clinical evidence. A recent multicenter cohort study involving 43,948 participants reported that individuals consuming oily fish at least twice per week exhibited an approximately 31% lower risk of developing cholelithiasis (hazard ratio = 0.69; 95% confidence interval: 0.54–0.88) (Chan et al. 2007). The biological mechanisms underlying this protective effect appear to be multifactorial; ω‐3 fatty acids in fish oil have been shown to enhance bile solubility and inhibit the formation and aggregation of cholesterol crystals (Zheng et al. 2025). In an interventional study, Shabanzadeh et al. observed that a 12‐week continuous fish oil supplementation regimen led to an 18.6% reduction in the cholesterol saturation index of bile (*p* < 0.001) (Shabanzadeh et al. 2017). Notably, a double‐blind, placebo‐controlled trial conducted by the Méndez‐Sánchez group demonstrated that EPA and DHA not only modulate bile composition but also markedly enhance gallbladder contractility (22.3% increase, *p* = 0.003) and reduce bile stasis (Méndez‐Sánchez et al. 2001). Among the identified genes, glucokinase regulatory protein (GCKR), which encodes the glucose kinase regulatory protein, serves an essential function in the regulation of hepatic glucose metabolism and lipid synthesis. Through GWAS, Ferkingstad et al. identified a significant association between GCKR variants and cholelithiasis risk (odds ratio = 1.24, *p* = 2.5 × 10^−11^) (Ferkingstad et al. 2018). This polymorphism has been found to impair the inhibitory function of GCKR, thereby promoting hepatic lipid synthesis and disrupting cholesterol metabolic balance. Further investigation by Stender et al. (2017), using protein structure analysis, revealed that GCKR variants alter their regulatory influence on glucokinase, leading to increased cholesterol secretion into bile. In clinical settings, Liu et al. reported that GCKR genotyping can effectively predict dietary intervention responses in cholelithiasis patients (Liu et al. 2017). FTO, another gene of interest, possesses polymorphisms that are markedly associated with gallstone formation (Ma et al. 2022). Lipid metabolism disorders induced by FTO overexpression have been implicated in increased cholesterol saturation and the promotion of cholesterol crystal formation in bile (Li et al. 2024). Additionally, FTO may contribute to inflammatory responses in the gallbladder wall by modulating inflammatory factor expression, thereby further facilitating gallstone development (Chen et al. 2023). The remaining five genes have received limited attention in the context of cholelithiasis and warrant further investigation.

Multiple clinical studies have demonstrated that raw vegetables rich in dietary fiber exert significant preventive effects against cholelithiasis. In a case–control study, Jessri and Rashidkhani (Jessri and Rashidkhani 2015) reported a significant inverse association between raw vegetable intake and cholelithiasis risk, with leafy green vegetables exhibiting the most pronounced protective effect. The abundant soluble fiber present in raw vegetables has been shown to reduce cholesterol saturation and inhibit the crystallization of cholesterol (Sun et al. 2024). From the perspective of genetic susceptibility, it has been proposed that high vegetable intake may regulate the expression of cholelithiasis‐related genes through epigenetic mechanisms. In their review, Lammert et al. (2016) indicated that dietary factors can modulate the expression of cholesterol transporter genes such as ABCG5/G8, which serve critical functions in the regulation of cholesterol secretion. Notably, interactions between dietary patterns and genetic factors have also been identified, as raw vegetable intake may attenuate the influence of specific gene polymorphisms on gallstone formation (Shabanzadeh et al. 2016). A meta‐analysis confirmed that an increase of 100 g in daily vegetable intake can reduce cholelithiasis risk by approximately 12% (Aune et al. 2017). Among the three previously identified genes, GCKR has been more extensively linked to cholelithiasis in prior studies, whereas the other two genes warrant further investigation in future research.

This study has several limitations. First, lifestyle‐related confounders correlated with diet (e.g., exercise, smoking) were not fully adjusted for, which may lead to partial attribution of non‐diet effects to diet. Second, current statistical methods cannot completely eliminate LD, and the lack of sensitivity analyses using alternative reference panels may influence the results. Third, sample overlap across GWAS datasets could bias genetic correlation estimates, despite LDSC corrections. Fourth, the study is limited to individuals of European ancestry, which restricts the generalizability of the findings; therefore, replication in more diverse cohorts should be prioritized in future research. Fifth, while MTAG increases statistical power, it assumes linear and homogeneous correlations, and potential non‐linear gene–diet interactions remain unexplored. Finally, the functional discussion of genes such as GCKR and APOE relies partly on evidence from other diseases, as direct studies on diet–gene–gallstone links are lacking. Future studies should incorporate pathway analyses based on dietary interventions, CRISPR‐based functional assays, and transcriptomic profiling in relevant tissues, combined with both in vitro and in vivo validation, to further elucidate these mechanisms.

## 5 Conclusion

In conclusion, this study elucidates the significant genetic overlap between cholelithiasis and four specific dietary patterns, providing molecular‐level evidence of associations. Multi‐dimensional genomics analyses identified several shared genetic loci. Although these findings are statistically significant, their biological relevance requires further confirmation through functional validation and clinical studies. If validated in future research, these loci may provide valuable clues for developing targeted hypotheses on the interaction between cholelithiasis and dietary patterns and may hold potential applications in personalized nutrition research.

## Author Contributions

**Endong Zheng:** conceptualization (equal), data curation (equal), writing – original draft (equal). **Bangzhun Cai:** methodology (equal), supervision (equal), writing – review and editing (equal). **Xiaowang Huang:** formal analysis (equal), methodology (equal), visualization (equal), writing – review and editing (equal).

## Ethics Statement

The authors have nothing to report.

## Conflicts of Interest

The authors declare no conflicts of interest.

## Supporting information


**Data S1:** fsn371052‐sup‐0001‐DataS1.xlsx.

## Data Availability

All the GWAS data and statistical software used in this study were publicly available (which can be accessed through the following URLs), and all the generated results in this study were provided in the main text and supplemental data. IEU database: https://gwas.mrcieu.ac.uk/. LDSC: https://github.com/bulik/ldsc, HDL: https://github.com/zhenin/HDL, LAVA: https://github.com/josefin‐werme/LAVA. ConjFDR: https://github.com/precimed/pleiofdr. MTAG: https://github.com/jonjala/mtag. FUMA: https://fuma.ctglab.nl. FinnGen: https://r12.finngen.fi/
